# Cervicothoracic Intradural Arachnoid Cyst Misdiagnosed as Motor Neuron Disease

**DOI:** 10.1155/2010/261657

**Published:** 2010-06-10

**Authors:** P. G. Sämann, H. Himmerich, T. Merl, C. Erös, M. B. Müller, J. C. Tonn, B. Buchwald

**Affiliations:** ^1^Max Planck Institute of Psychiatry, Kraepelinstraße 2-10, 80804 Munich, Germany; ^2^Claussen-Simon-Endowed Professorship, Department of Psychiatry and Psychotherapy, University of Leipzig, Semmelweisstraße 10, 04103 Leipzig, Germany; ^3^Department of Neurosurgery, Klinikum Großhadern, Ludwig Maximilians University, Marchioninistraße 15, 81377 Munich, Germany

## Abstract

Recognizing syndromes which mimic ALS is crucial both to avoid giving this diagnosis erroneously and since there may be appropriate treatments. We report a 63-year-old woman diagnosed with possible ALS five years ago based on upper and lower motor neuron signs with typical electrophysiology and normal cranial MRI. At reassessment, spinal MRI revealed a cervicothoracic cyst with cord compression that was successfully treated neurosurgically. Histopathology confirmed an arachnoid origin as suspected from MRI. Spinal cysts may mimic ALS and need to be thoroughly excluded by appropriate imaging.

## 1. Introduction

Amyotrophic lateral sclerosis (ALS) is a subset of motor neuron disease in adults and usually causes death within a few years [1]. Definite diagnosis requires the presence of both upper and lower motor neuron signs in the bulbar, arm and leg musculature with clear evidence of progression. At initial presentation symptoms are often more focal, and especially in the early phase making the diagnosis of ALS challenging even for experienced neurologists. Electrophysiological investigations are necessary to differentiate ALS from potentially treatable polyneuropathies and myopathies [1]. Structural cranial or spinal pathologies should be excluded by magnetic resonance imaging (MRI) [1]. The misdiagnosis of ALS has far-reaching implications, for patients and their families given such a diagnosis, and because potentially curative treatments exist for certain ALS mimic syndromes. Overall, the Escorial criteria are conservative and include a criterion “progressive spread of symptoms or signs within a region or to other regions” to give sufficient weight to longitudinal observations [2]. We report a 63-year-old woman with major depression after being diagnosed with ALS for five years. At reassessment, renewed MRI revealed a cervicothoracic cyst which was successfully treated neurosurgically.

## 2. Case Report

A 63-year-old woman was admitted to the clinic of the Max Planck Institute of Psychiatry with major depression. Her onset of depressed mood had been gradual after being diagnosed with ALS five years ago. The patient had noted intermittent cramp-like sensations round her chest and a weak, numb neck pain. These symptoms had been fluctuating but not clearly progressive, for about two years when she was referred to a neurologist. File review showed that clinical presentation at that time already comprised objective weakness, atrophy and fasciculations in the upper limb muscles, along with extremely brisk tendon reflexes. No unequivocal sensory deficit such as hypesthesia or dysesthesia was documented at that time. Electromyography showed abnormal spontaneous activity in the right deltoid muscle, abducens pollicis brevis and palmar interossei muscles, and signs of bilateral chronic denervation in the upper limb which in combination with a normal cranial MRI scan was interpreted as motor neuron disease by several neurologists. In addition, a bilateral carpal tunnel syndrome was presumed on the basis of increased distal motor latencies of the median nerve. About two years later she was diagnosed with “possible ALS” according to El Escorial criteria [2].

Neurological examination at the time of admission to our clinic (about 7 years after the first symptoms) revealed normal cranial nerves with no signs of bulbar dysfunction and no fasciculations or weakness of facial, tongue, or neck muscles. Distinct muscle atrophy along with mild fasciculations of the upper limbs with right-sided, proximal accentuation was noted, particularly in the supraspinatus, deltoid and biceps muscles, and the finger extensors with motor strength reduced to 3-4/5. At the lower limbs, motor strength was of 4/5 of foot extensors, but no obvious atrophy or fasciculations was found. Weakness of the proximal lower limb muscles was suspected since she had difficulties in rising from sitting. She was able to walk independently but showed gait ataxia. Reduced vibration proprioception of all limbs and hyper- and dysesthesia of the shoulders and proximal arms were found. Deep tendon reflexes were symmetric, yet, extremely brisk, Babinski's signs were absent and superficial abdominal reflexes were retained. There were no symptoms of bladder or bowel dysfunction, but she reported neck pain and numbness and clumsiness of both arms.

**Figure 1:**
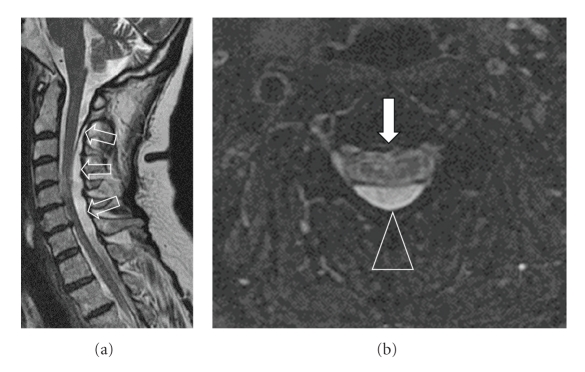
MRI findings at reassessment. Cystic cavity extending from cervicocranial junction to the T4 vertebral body with myelon displacement ((a)-*open arrows*). A thick membrane separated the T2-hyperintense mass ((b)-*triangle*) from the spinal cord ((a)-*long arrow*). Note myelopathy signal at vertebral body heights C4/5 and disc heights C6/7 on T2-weighted (a) and T2*-weighted images ((b)-*filled arrow*).

Proximal muscle atrophy in the arms together with sensory dysfunction and pain led anew to the suspicion of spinal pathology. Cervical MRI revealed a cyst in the dorsal intradural-extramedullary space of the cervicothoracic region. The lesion displaced the cervical spinal cord, and myelopathy was noted (Figure 1). Myelography and postmyelography CT were confirmative, and the patient was presented to the neurosurgeon.

Intraoperatively, elevated pressure within the cyst was found. Cyst membranes were partially removed with fenestration of the remaining parts via a right C6/C7 hemilaminectomy. Histopathology confirmed the arachnoid origin of the cyst.

Postoperatively, the patient reported major relief of her neck pain and regained strength in her arms. One year later, motor and sensory dysfunctions were minimal and no acute denervation was recorded on electromyography. After stabilisation of her general condition she also recovered from depression.

## 3. Comment

The cervicothoracic cyst in our patient had mimicked ALS symptoms as progressive amyotrophy and fasciculations along with brisk tendon reflexes and electrophysiological signs of acute and chronic denervation. Only one case considering a spinal cyst as a differential diagnosis to ALS has been reported: Korosue et al. [3] described a 49-year-old man with an intramedullary cyst of the conus medullaris, who presented with progressive amyotrophy of the lower limbs, clinically resembling ALS. Like our patient, this patient experienced considerable improvement after surgery.

Arachnoid cysts are rare outpouchings of the arachnoid lining and may be congenital, secondary to trauma or infection, or acquired, though idiopathic [4]. To date, approximately 100 cases have been reported, with the majority (95%) occurring in the thoracic or cervical section, only exceptionally exceeding a length of three vertebral bodies [2]. MRI is the diagnostic procedure of first choice [5, 6]. Some lesions may elude diagnosis due to the similar MRI signal of the cyst compared with adjacent subarachnoid spaces. Myelography and myelocomputed tomography may occasionally be more sensitive and provide information on CSF flow dynamics [6]. In our case, neither the cystic cavity nor its membrane or mass effect was detected by the first radiological workup that comprised cranial but no spinal MRI.

The hint for spinal pathology came from insistent focal symptoms without true spreading over time. Only about 10% of ALS patients present solely with amyotrophy in limb muscles; widespread involvement usually follows. Weakness and atrophy at onset are more frequent in the upper limbs than in the lower limbs or bulbar muscles [7]. Furthermore, proximal muscles, such as the shoulders, are less commonly involved at the beginning [7]. ALS patients may suffer from neck pain, secondary to overstraining, when pain radiation is usually to the shoulders, rather than down the arms, which can be useful in differentiating ALS from myelopathy [1]. Somatosensory symptoms occur in about 10% of ALS cases but tend to be mild, short lived, and more apparent in the early stages [7]. In our case, the patient had experienced intermittent, cramp-like sensations of the chest wall as an early complaint. Retrospectively, and in the context of analysing potential misclassifications, this type of pain syndrome should have raised the suspicion of a spinal pathology.

It has been estimated that up to 10% of patients initially diagnosed as ALS are ultimately rediagnosed as having a different disease. Importantly, for about half of these patients potentially curative treatments exist [8]. This latter percentage was estimated in an investigation of 552 registered ALS patients reported by Davenport et al.: in this report, specific treatment options were identified for 23 patients, half of 46 for which an alternative diagnosis was made. The predominant reasons which led to a diagnostic revision were failure of symptom progression, development of atypical clinical features for MND, and investigation results [8]. More than 90% of patients with ALS mimic syndromes reported by Traynor et al. had presented with symptoms referable to the limbs and without signs of bulbar involvement, similar to our case [7]. Two of 32 patients had noncompressive myelopathy and one had cervical spondylitic myelopathy [7], thus cervicothoracic cysts probably represent rare causes of ALS mimic syndromes. In terms of prognosis, our case adds to reports on giant cervicothoracic cysts with excellent outcome after surgery [3, 9–12]. The delayed detection of a spinal pathology in our case alerts to strict adherence to the accepted Escorial scheme that defines the “lack of other neuroimaging evidence of other disease processes” as one of five essential criteria [2].

Another essential precondition for a diagnosis of ALS is the “progressive spread of symptoms or signs within a region or to other regions, as determined by history or examination” [2]. This criterion is pivotal since progression is typical for true motor neuron disease. In fact, our patient showed local progression in terms of increasing muscle atrophy, weakness of the upper limbs, and worsening of gait ataxia, compatible with an increasing space-occupying effect of the cyst. However, no unequivocal spread of lower motor neuron signs to the lower limbs or the brainstem was noted.

In conclusion, cervivothoracic arachnoid cysts could be added to the group of treatable ALS mimic syndromes. Careful longitudinal clinical assessment, complete application of diagnostic measures including spinal imaging, and strict adherence to recommended diagnostic criteria of ALS as a minimum standard are essential. Specific attention to the possibility of ALS mimic syndromes is needed when there is clinical evidence of neuronal involvement outside the motor system, an unusual distribution of muscle atrophy with lack of spreading to other regions or persisting sensory or pain symptoms. Such clinical features should alert the physician to question the diagnosis and take further diagnostic measures.
